# Integrating advanced practice nurses into the health system: views from nurses and healthcare managers – a qualitative study

**DOI:** 10.1186/s12912-026-04427-z

**Published:** 2026-02-20

**Authors:** Ingrida Qamar, Jekaterina Šteinmiller, Natalja Istomina

**Affiliations:** Department of Midwifery and Nursing, Institute of Health Sciences, Faculty of Medicine of Vilnius University, Universiteto St. 3, Vilnius, LT- 01513 Lithuania

**Keywords:** Advanced Practice Nurses, Healthcare managers, Quality of care, Lithuanian health system, Qualitative study

## Abstract

**Background:**

Despite the pivotal role that nurses occupy within the Lithuanian healthcare system, the potential of Advanced Practice Nurses (APNs) remains largely unrealized due to the absence of clearly defined formal roles and structured pathways supporting autonomous practice and full use of advanced competencies. This study aimed to examine the perspectives of selected nurses and healthcare managers on the integration of APNs into the health system.

**Methods:**

A qualitative descriptive design employing semi-structured interviews was utilised in Lithuania with Advanced Practice Nurses (*n* = 6) and healthcare managers (*n* = 6). Participants were recruited based on inclusion criteria. The interviews covered six main topics. The average duration of the interviews was 67.5 min. Data were analyzed using thematic analysis guided by a qualitative descriptive framework, informed by role theory and health workforce integration perspectives.

**Results:**

The analysis yielded twelve themes each for the nurses and healthcare managers. The results indicated that Advanced Practice Nurses and healthcare managers have differing perspectives on how Advanced Practice Nurses should be integrated, yet both regard it as a necessary evolution of the health system. Motivations were both personal and professional, driven by a commitment to holistic, patient-centred care and the desire to apply advanced clinical knowledge autonomously. Managers perceived successful integration as dependent on role clarity, mutual trust, and professional autonomy. Integration barriers are chiefly structural and cultural—unclear legislation, insufficient funding, low public awareness, and professional resistance—but can be addressed through policy reform, education, public engagement, and investment in collaborative models.

**Conclusions:**

Advanced Practice Nurses are perceived by nurses and healthcare managers as having the potential to strengthen healthcare delivery, particularly in supporting primary care, managing chronic conditions, and improving access in underserved regions. Although APNs demonstrate strong motivation to expand their roles, this potential is currently constrained by limited institutional pathways, role clarity, and recognition. Further national-level research in Lithuania is needed to evaluate the clinical, organizational, and economic impact of Advanced Practice Nurse–led care.

**Clinical trial number:**

Not applicable.

**Supplementary Information:**

The online version contains supplementary material available at 10.1186/s12912-026-04427-z.

## Background

As healthcare systems grow more complex, many countries are expanding the roles of nurses to enhance access, improve outcomes, and increase system efficiency [1]. Advanced Practice Nurses (APNs)—including Nurse Practitioners, Clinical Nurse Specialists, Nurse Midwives, and Nurse Anesthetists—are increasingly acknowledged for their expert knowledge, clinical autonomy, and decision-making capabilities, usually supported by a master’s degree or higher [2, 3].

While the concept of APNs is broadly accepted, implementation differs due to variations in legislation, system readiness, and professional recognition. The International Council of Nurses (ICN) provides global benchmarks, recommending national regulation and master’s-level education incorporating advanced assessment, pharmacology, pathophysiology, clinical reasoning, leadership, and research [4–6]. In addition, the Participatory, Evidence-based, Patient-focused Process for Advanced Practice Nursing (PEPPA) framework [7], is internationally recognised as a guiding framework for the systematic development, implementation, and evaluation of APN roles. The PEPPA framework emphasises stakeholder engagement, alignment of population health needs with role competencies, and the integration of education, regulation, and organisational support, all of which are critical for sustainable APN implementation [7].

In countries like the U.S. and Canada, APNs are educated through accredited graduate programs that include simulation, interprofessional training, and clinical preceptorships under national oversight [8, 9]. Austria emphasizes outcome-based education [10], while Spain and Switzerland are piloting multidisciplinary programs integrating nursing science, clinical practice, and health policy [11, 12].

Countries with mature APN models report better outcomes, higher patient satisfaction, and increased cost-effectiveness [13]. France, for example, has improved access and responsiveness following the legal adoption of APNs in 2018 [14]. Switzerland’s APNs play key roles in meeting primary care demands, although challenges like role clarity persist [11].

Lithuania mirrors trends seen in other Central and Eastern European countries, where APN development is still in its infancy and inconsistent [15]. Although advanced nursing is offered at the bachelor’s level, formal postgraduate APN pathways are only beginning to emerge [4]. This situation creates a gap between current academic preparation and the advanced clinical competencies expected of APNs, particularly in relation to autonomous clinical decision-making, leadership, and responsibility for complex patient care. A lack of structured, ICN-aligned curricula prevents nurses from acquiring the competencies and credentials needed for independent practice [5]. From a PEPPA perspective, this misalignment between education, clinical expectations, and regulatory support represents a critical barrier to effective role implementation [7]. Additionally, program quality is constrained by limited placements and preceptors [9].

Despite these limitations, pilot initiatives in home-based care and integrated primary care are testing expanded roles for nurses, particularly in chronic care [16, 17]. Universities and professional expert groups are collaborating to launch APN-focused educational offerings. However, these initiatives remain fragmented and insufficiently embedded within a national framework that would ensure consistency, competency alignment, and role sustainability.

Several barriers hinder APN integration in Lithuania. Key among these is the absence of a national regulatory framework. Unlike countries with mature APN systems that legally define and protect APN roles, Lithuania lacks formal definitions and title protection [9, 18, 19]. Consequently, nurses often perform advanced tasks without official recognition or consistent practice standards. The absence of clear role definition and regulatory endorsement undermines role legitimacy and limits system-level acceptance [7].

Physician resistance also remains a significant obstacle, as doctors may view advanced nursing roles as encroachment, especially in hierarchical systems [15, 20–22]. Some Lithuanian physicians express doubts about nurses’ ability to practice autonomously, largely due to limited awareness of APN competencies and traditional team dynamics [23, 24]. Conversely, countries like the UK, Canada, and Switzerland show that clear role definitions, trust-building, and institutional support can enable collaborative, team-based APN models [11, 25].

Public and professional awareness is another major challenge. Without recognition of APN competencies, patients and colleagues may undervalue these roles [12, 26]. However, international evidence suggests that once patients experience APN-led care, acceptance and support grow [11, 12, 27].

The Lithuanian Ministry of Health has recognized the need to expand nursing roles to alleviate physician shortages, particularly in underserved regions. The 2024 ‘Action Plan to Reduce the Shortage of Doctors’ (formally the Health Professional Attraction and Retention Action Plan 2024–2029) includes explicit provisions to enhance the training and retention of nurses, reflecting a strategic shift toward extending nursing competencies [28]. Nevertheless, without parallel development of APN-specific regulation, education standards, and competency frameworks, policy intentions may not translate into effective role implementation [29–32].

Pilot projects like Integrated Team-Based Home Care have demonstrated the feasibility and benefits of advanced roles despite lacking regulatory frameworks [33]. Comparatively, Nordic countries such as Finland, Sweden, and Norway have established clear APN regulations, protected titles, and ICN-aligned master’s programs [4, 15, 34, 35]. Finland’s nurse practitioners function independently in both primary care and leadership positions, while Sweden implements national frameworks across care settings [36, 37].

To advance APN integration, Lithuania must invest in education, regulation, faculty, and fair compensation [20]. Though APNs can improve chronic care and reduce hospital readmissions [38], initial investment is difficult to secure without robust pilot data or external funding [13]. Role recognition, institutional support, and public awareness are essential to prevent skilled nurses from feeling undervalued and underutilized [4, 39–41].

Healthcare managers in Lithuania support APN implementation but stress the need for clear role definitions and structured postgraduate training. While nurses are eager to take on more responsibility, wider acceptance will depend on sustained awareness-raising and trust-building efforts. International collaboration, particularly with Nordic countries, could help accelerate development [42, 43].

Lithuania’s interest in APN roles aligns with global efforts to optimize nursing practice to meet workforce demands and improve care delivery. While the potential is evident, the absence of formal regulation, education, and recognition remains a barrier. With targeted investment and strategic alignment with ICN standards, Lithuania can harness the full potential of Advanced Practice Nursing to strengthen its healthcare system.

## Methods

### Aim and research questions

The aim of the study was to examine the perspectives of selected nurses and healthcare managers on the integration of Advanced Practice Nurses into health system.

To achieve this aim, the following research questions were formulated:

1. What is the perceived need for advanced practice nurses in the Lithuanian health system from the perspectives of nurses and healthcare managers?
2. What motivates advanced practice nurses within the Lithuanian health system according to nurses and healthcare managers?
3. What problems and possible solutions do nurses and healthcare managers identify regarding the integration of advanced practice nurses into the Lithuanian health system?

### Study design and setting

This study uses exploratory, qualitative interpretative design and adopts an interpretivist paradigm and a phenomenological approach to explore nurses’ perceptions, recognising that meaning is constructed through their lived experiences [44]. This design was chosen because it allows flexibility in data collection, provides rich and detailed qualitative data, and is particularly suitable for gaining comprehensive insights into participants’ personal experiences, perceptions, and attitudes related to the topic [45].

### Sampling and study participants

This study was conducted in healthcare institutions providing emergency care, anaesthesia and intensive care, and primary healthcare services. Interviews were carried out between February - March, 2025. Participants were recruited using purposive sampling [46], based on their professional roles, experience, and relevance to the research objectives concerning the integration of APNs into healthcare settings. Initial contact with the potential participants was made through formal emails and institutional channels. Upon expressing interest, participants were provided detailed information about the research objectives, procedures, and ethical considerations. Written informed consent was obtained from each participant prior to conducting the interviews [47]. Efforts were made to accommodate participants’ schedules, ensuring convenience, privacy, and comfort during data collection.

Inclusion criteria of the APN:

- Having advance practice nursing degree in the field of emergency care, anaesthesia and intensive care, or primary healthcare settings.
- Having an active advance practice nursing licence.
- Willing and able to provide informed consent and participate in recorded interviews.

Exclusion Criteria of the APN:

- Not actively involved in clinical work as an APN.
- Unable or unwilling to provide informed consent or to participate in recorded interviews.

Inclusion criteria of the healthcare managers:

- Holding positions of director, head nurse, or other administrative roles directly involved in the decision-making related to nursing practice and integration of APNs.
- Willing and able to provide informed consent and participate in recorded semi-structured interviews.

Exclusion criteria of the healthcare managers:

- Not directly involved in the administrative processes concerning APN integration.
- Unable or unwilling to provide informed consent or to participate in recorded interviews.

Participants were purposively selected due to their direct roles, professional experience, and in-depth insight into the integration and management of advanced practice nursing within their respective institutions. Advanced Practice Nurses were eligible if they held a relevant APN degree (in emergency care, anaesthesia and intensive care, or primary healthcare), possessed an active APN license, and consented to participate in recorded interviews. Healthcare managers were included if they held leadership or administrative roles (e.g., director, head nurse) directly involved in decision-making related to APN integration. This highly specific and information-rich sample supported the principle of information power, whereby a smaller number of participants is sufficient when the study aim is focused, participants possess substantial experiential knowledge, and the quality of dialogue is strong. Accordingly, the sample size (*n* = 12) was considered adequate to generate meaningful and robust qualitative findings relevant to the study objectives.

### Data collection

Data were collected using a semi-structured interview guide [48], which was specifically developed for this study, has not been previously published elsewhere, and was designed to explore the experiences, perceptions, and expectations regarding the integration of APNs into the Lithuanian healthcare system. An English-language version of the interview guide developed for this study has been added as a supplementary file.

The guide was drafted by the first author based on an analysis of evidence-based literature to ensure alignment with the research objectives and academic rigor and was subsequently approved by the co-authors to ensure clarity, coherence, and relevance to the study’s aims.

Two tailored versions of the guide were created for different participant groups. One for APNs focusing on their clinical practice, motivation, and vision for professional development. Other for healthcare managers, addressing institutional strategies, policy considerations, and organizational readiness for APN integration. The interview guide consisted of questions organized into key thematic areas, including:

- Professional roles and related responsibilities.
- Perceived healthcare needs and APN contributions.
- Barriers and challenges to APN integration.
- Motivation and professional career development.
- Support at the organizational and systemic levels.
- Anticipated developments in future healthcare delivery models.

Participants were explained the purpose of the study, and the importance of their shared experience was emphasized. In addition, they were informed that the participation in the study is voluntary, and they can withdraw at any stage of research. Before the interviews, informed consent forms were signed, then digitised after the interviews and stored in a secure cloud server of the university, while the paper versions were destroyed using a paper shredder.

All interviews were conducted by the first author, individually, face-to-face, in Lithuanian, recorded with informed consent using a secure research-designated mobile device. The duration of interviews was from 45 to 90 min.

After transcribing the interviews, the participants were coded, and all potentially identifiable data was removed. After transcription, all recordings were deleted. The resulting database was stored on the university’s secure cloud server, with access restricted to the first author.

### Data analysis

Data collection continued until thematic saturation was achieved, meaning that no new themes or relevant insights emerged in the later interviews within each participant group. Data analysis was conducted using reflexive thematic analysis. Initially, all recorded interviews were manually transcribed by the researcher. Transcripts were carefully reviewed multiple times to ensure accuracy and familiarity with the data by all the authors. The thematic analysis process involved the following stages [49]:

#### Familiarization

Repeated reading of transcripts to understand participants’ perspectives thoroughly.

#### Coding

Relevant segments of text were identified and assigned initial codes based on repeated ideas and concepts directly linked to the research questions.

#### Categorization and thematic analysis

Codes were grouped into broader categories and subcategories, facilitating the identification of main themes and patterns.

#### Interpretation and description

Identified themes were interpreted and described, ensuring the participants’ perspectives regarding the integration of Advanced Practice Nurses into healthcare settings were accurately represented. Individual participant quotations will be included to illustrate key findings.

## Results

The study included six APNs—two from each specialty working at the data collection in emergency care (*n* = 2), anaesthesia and intensive care (*n* = 2), and primary healthcare (*n* = 2) were involved. As well as 6 healthcare managers who contributed as health institutions directors (*n* = 2), head nurses (*n* = 2), and health care institutions deputy directors (*n* = 2).

### Overview of study participants

Participants were purposefully selected for their experience and direct involvement in APN integration, ensuring diverse perspectives and rich qualitative data. The interviewed nurses reported between 5 and 12 years of experience as nurse practitioners and 1–3 years as advanced practice nurses. Among the healthcare managers, the directors had 2–3 years of experience in their current roles, whereas the deputy directors reported 5 and 7 years of experience, respectively. The head nurses had accumulated 4 and 8 years of experience in their positions.

The interviews highlighted key themes and subthemes pertaining to nurses’ perceptions in Table 1.

**Table 1:** Perceptions of APNs: the main themes and subthemes identified

Theme	Subtheme
Current Roles and Evolving Responsibilities, and Professional Boundaries	• Formal role remains as General Practice Nurse despite advanced qualifications. • Gradual shift in physician perception of nurse capabilities. • Professional Interaction and Role Boundaries with Physicians.
Perceived healthcare needs and APN contributions	• Addressing diagnostic delays, limited test access, and chronic disease management. • Reducing waiting times and enhancing patient access, especially in rural areas.
Barriers and challenges to APN integration	• Lack of legal authority and restrictive policies. • Resistance from older-generation healthcare professionals and lack of public awareness.
Professional motivation and career development	• Desire for greater autonomy and recognition of clinical expertise. • Decline in motivation due to limited system-level support.
Organizational and system-level support	• Need for mentorship, educational programs, and consultation spaces. • Importance of management and policy backing for APN roles.
Expectations for future healthcare delivery models	• APNs as public health leaders and contributors to preventative care. • Integration leading to more responsive, flexible, and sustainable health systems.

### Current roles and evolving responsibilities, and professional boundaries

The APNs described a wide array of responsibilities in their current practice, including delivering preventive care, administering immunizations, performing home visits, and coordinating care with other professionals. Despite their qualifications and experience, most participants stated that their formal roles remain confined to those of General Practice Nurses (GPNs).

After completing my master’s studies, my role has not changed… I still work as a General Practice Nurse, with the same responsibilities, but now with broader knowledge. – Nurse 6

Other noted a slow but noticeable shift in their responsibilities, where physicians are gradually beginning to appreciate and rely on nurses’ knowledge and perspectives. This ongoing transition illustrates both the stagnation and the potential for growth in the professional scope of APNs, particularly in settings where systemic support is lacking.

In my daily practice, my main role is still primarily following physicians’ orders and assisting them. However, I have noticed that doctors’ attitudes are gradually changing…. – Nurse 3

Several APNs described their interactions with physicians as predominantly hierarchical, characterised by limited autonomy and role ambiguity. Although collaboration was reported, decision-making authority largely remained with physicians, reinforcing traditional professional boundaries. Some nurses noted gradual improvement in mutual trust; however, they continued to perceive their role as subordinate rather than complementary.

Even with a master’s degree, I still need confirmation from the doctor. My opinion is heard, but the final decision is not mine. – Nurse 4

Doctors are slowly starting to trust us, but the system still treats us as assistants, not partners. – Nurse 2

### Anticipated impact of APN integration

Participants were unanimous in their belief that APN integration would significantly enhance their roles, offering greater independence in clinical decision-making, patient assessment, and chronic disease management. There was a strong belief that such integration would not only benefit individual nurses but also the wider healthcare system.

With the integration of Advanced Practice Nurses into the healthcare system, my role will become more autonomous, with greater responsibility and influence in patient care decision-making. – Nurse 1

The APN role was envisioned as bridging gaps in accessibility, especially in rural and underserved regions, while supporting the redistribution of tasks traditionally handled by physicians. Such expanded responsibilities are seen as a way to increase efficiency, reduce waiting times, and ensure that more complex cases receive appropriate attention from physicians.

We could manage less difficult patients… APNs could take over home visits and even handle emergency patients with flu-like symptoms. – Nurse 2

### Challenges in current practice addressable by APNs

Participants reported several systemic and operational barriers that limit effective care delivery. Many challenges—such as delayed diagnostic decisions, the inability to prescribe or order routine tests, and long waiting times—were seen as solvable through expanded APN roles.

We can’t order certain tests… patients must be sent to the GP just to order harmless tests like ferritin or vitamin D. – Nurse 2

If a patient comes in for a blood transfusion, an APN could order it without waiting for a physician… saving valuable time. – Nurse 3

Several nurses also stressed that APNs could act swiftly in acute situations, preventing unnecessary delays and hospitalizations.

There are cases where a nurse’s assessment would be sufficient to provide needed services, but due to lack of legal authority, we are limited. – Nurse 6

### Healthcare needs addressed by APNs

The nurses identified major public health and systemic challenges that could be addressed through the inclusion of APNs. These included access to care in remote areas, increasing numbers of patients with chronic conditions, and insufficient patient education.

APNs could monitor patients with chronic diseases… and implement health promotion programs that reduce disparities and improve overall public health. – Nurse 1

In the future, APNs could even replace GPs in remote areas where there are staff shortages. – Nurse 2

Other suggestions included structuring APN roles around chronic disease panels, where each APN would be responsible for the long-term management of a specific patient group.

### Motivations to pursue the APN role

Motivations were both personal and professional. Participants were driven by a desire to provide holistic, patient-centred care and to apply their expanded clinical knowledge in a more autonomous setting.

It’s thrilling when your patients come back to you instead of registering with the GP—it shows trust and independence. – Nurse 2

I believe that advanced practice functions allow nurses to fully realize their potential—not just following doctors’ orders, but actively participating in decision-making, prevention, and education. – Nurse 1

Some nurses described internal barriers to motivation, particularly in settings where their potential remains underutilized.

At the moment, I feel a significant drop in motivation because… I don’t see any real prospects for APNs in Lithuania due to lack of regulation and support. – Nurse 6

### Professional development and career satisfaction

Nurses emphasized that APN roles promote continuous learning and create opportunities for advancement into teaching, management, and research roles. They expressed a strong sense of purpose in being able to impact patient care directly.

The APN role opens new paths in clinical practice and leadership. It’s empowering to see how your knowledge can shape outcomes. – Nurse 5

Nonetheless, they also stressed that without institutional recognition and proper job structures, the benefits of advanced education remain theoretical.

To enhance professional development, more APN positions need to be created, and management must trust in APN competencies. – Nurse 3

### Barriers to APN integration

Numerous challenges were identified that hinder the integration of APNs, including unclear legal frameworks, scepticism from colleagues, and lack of awareness among the public. In addition to regulatory barriers, interprofessional dynamics played a significant role in limiting APN integration. Several participants described implicit resistance from physicians related to unclear role boundaries and concerns over accountability, which constrained collaborative practice.

There’s still a lack of legal frameworks, mistrust from doctors, and unclear role definitions. – Nurse 1

I see a negative attitude from older-generation nurses and doctors… and scepticism from patients. – Nurse 6

Structural barriers like restrictive laws and inadequate compensation models were also noted.

Laws in some cases are very restrictive… they should change to give more freedom to APNs. – Nurse 2

### Systemic changes needed for APN integration

Participants proposed clear and actionable changes necessary for successful integration. These included:

- Establishing legal and regulatory clarity.
- Developing APN job descriptions across all levels of care.
- Creating financial incentives and reimbursement systems.
- Launching public education campaigns about APN roles.

First, a clear legal framework must be established to define APN competencies… this would avoid confusion and ensure safe, high-quality service delivery. – Nurse 1.

There needs to be clear regulations… and a defined set of job requirements for APN positions. – Nurse 4

### Support and resources required

Support systems were seen as vital, especially during the early implementation phase. Nurses stressed the need for mentorship, access to continuous education, administrative support, and physical infrastructure like consultation rooms. The role of healthcare institutions in mentoring and promoting APNs was emphasized as a critical factor in successful transition.

Support from administration is very important, and collaboration with GPs is also essential. – Nurse 2

Educational institutions should provide practical training and ensure programs meet real-world demands. – Nurse 5

### Expected impact on the healthcare system

All participants agreed that APNs would play a crucial role in improving the healthcare system’s responsiveness, especially amid physician shortages and increasing chronic disease prevalence.

APNs could significantly improve patient health outcomes… they can dedicate more time to education and consultations. – Nurse 5

They would improve system flexibility, accessibility, and sustainability. – Nurse 1

They envisioned APNs not only as care providers but also as educators, policy influencers, and leaders in public health.

APNs can become not just care providers, but also leaders in public health… their potential is still underutilized in Lithuania. – Nurse 1

Many APNs described experiencing a professional identity transition marked by tension between their advanced education and their constrained clinical role. Participants reported uncertainty regarding their professional identity, feeling positioned between general nursing and medicine without formal recognition of their advanced status.

Sometimes I feel like I am expected to be more than a nurse, but I am not allowed to be fully independent either. – Nurse 6

The interviews revealed the following themes and subthemes related to healthcare managers’ perceptions presented in Table 2.

**Table 2:** Perceptions of healthcare managers: the main themes and subthemes identified

Theme	Sub-theme
Current professional roles and responsibilities	• APNs understood as independent decision-makers and care providers. • Capability to prescribe, refer, and manage chronic conditions.
Perceived healthcare needs and APN contributions	• Relieving physician workload and improving care access. • Enhancing patient care continuity, especially in underserved areas.
Barriers and challenges to APN integration	• Legal ambiguity, unclear scope, and interprofessional resistance. • Public misunderstanding and cultural resistance in rural areas.
Professional motivation and career development	• Support for APNs as a modern evolution of skilled nursing practice
Organizational and system-level support	• Clear job roles, institutional support, fair compensation. • Need for national legislation, education campaigns, and structural planning.
Expectations for future healthcare delivery models	• Improving system efficiency and expanding care access. • Phased integration, increased public trust, and interdisciplinary cooperation. • Defined success as clarity, autonomy, and measurable health outcomes.

### Understanding of the APN role

Healthcare managers (HM) showed clear support for the APN role, highlighting their broad clinical skills, independent decision-making, and ability to connect physicians and nurses. While most healthcare managers expressed strong support for APN autonomy, some descriptions suggested ambivalence, implicitly positioning APNs either as physician substitutes or as highly skilled assistants rather than as a distinct professional role. One saw APNs as autonomous healthcare professionals, not just assistants.

The APN role is important… a specialist with broader competencies, having sufficient knowledge and skills to examine patients, make decisions, and act independently. – HM 1

APNs are practically like doctors… They work independently, in separate offices from doctors, consulting physicians only in emergency situations. – HM 4

I would say advance practice nurse is a highly trained nurse with advanced clinical skills and the ability to work independently in certain areas, such as diagnosis, prescribing, and chronic disease management. – HM 6

One HM reinforced this vision by highlighting that APNs could take over various tasks traditionally performed by physicians, such as issuing prescriptions, ordering and interpreting tests, and referring patients to specialists.

APNs could support the doctor, prescribe medications, order tests, interpret results, and write referrals. – HM 3

### Perceived contribution of APNs to patient care

HMs uniformly agreed that APNs could make substantial contributions to patient care, particularly by relieving physician workload and improving access to healthcare services. They envisioned APNs as key contributors to both acute and chronic patient management. These reflections underscore the potential for APNs to expand healthcare system capacity without compromising care quality.

They would relieve the burden on doctors, take over part of their functions, and ensure faster access to care. – HM 1

In our institution… APNs would reduce the workload of both doctors and nurses and should be able to provide many services independently. – HM 3

Some highlighted their role in addressing care gaps when doctors are unavailable, especially in rural or underserved regions.

In outpatient clinics, it’s nearly impossible to get doctors to work. This is another niche for APNs—to manage routine cases and refer difficult ones. – HM 4

### Areas of significant impact

When asked about specific areas or departments where APNs could have the greatest influence, HMs pointed to primary healthcare, chronic disease management, outpatient services, emergency rooms and even anaesthesiology.

At the primary care level… APNs could independently take over a large share of work, especially in chronic disease management. – HM 5

I think APNs would be especially effective in family medicine departments. – HM 3

Especially in primary care, emergency departments, and home-based care programs APN’s can have significant impact in improved and timely care. – HM 6

This broad applicability suggests APNs could help optimize patient flow and improve outcomes across care levels, provided the system enables their full integration.

#### Healthcare needs addressed by APNs

The healthcare system in Lithuania faces multiple pressures, including prevalence of chronic diseases, physician shortages, and regional disparities. HMs believed APNs are uniquely positioned to help alleviate these challenges.

Independent consultations, prescription renewals, monitoring of patients with multiple chronic conditions—APNs could cover all of these needs. – HM 1

The most urgent needs are related to physician shortages, particularly in regional hospitals, and the growing burden of chronic diseases. APNs could help address all these issues. – HM 6

HMs also emphasized the importance of preventive health initiatives and patient education, both of which fall naturally within the scope of advanced nursing practice. However, there was concern over the current ambiguity in legal definitions of the APN role.

Currently, the law defines the role very broadly but without clarity. That’s just my opinion. – HM 3

### Impact on organizational efficiency and outcomes

HMs highlighted APNs’ potential to increase healthcare delivery efficiency by handling routine cases independently and allowing physicians to focus on complex cases.

If APNs handle straightforward consultations independently, it will speed up access to specialists for urgent or complex cases. – HM 5

Some cautioned that the effect on patient wait times would depend on how extensively APNs are implemented.

If there were at least four APNs in a large facility, yes—it would make a difference. But one APN might not change much. – HM3

Nonetheless, the overall sentiment was that APNs would improve service coordination and responsiveness.

#### Resources, funding, and integration support

Successful integration requires systemic and institutional support. HMs emphasized the need for clear legal definitions, proper workplace setup, access to modern tools, and appropriate salary structures.

First, clearly define duties, rights, and responsibilities, and update the legal framework. – HM 5

Support from hospital management is essential, along with ministerial orders and clear job roles. – HM 3

Financial sustainability was a recurrent concern. While APN roles are seen as cost-effective in the long run, initial implementation and funding strategies remain unresolved.

Maintaining an APN position would cost less than a family doctor or specialist… funds could be used for more diagnostics and tools. – HM 2

#### Anticipated challenges

Key challenges included lack of trust from existing medical staff, unclear regulations, and public perception. Resistance could arise from both physicians and general nurses if roles and responsibilities are not clearly communicated.

Doctors may resist if they’re held accountable for APN errors. Nurses may resist due to wage gaps. – HM 1

The biggest challenge will be role clarity—differentiating APNs from general nurses and physicians. – HM 6

The need for public education was repeatedly stressed, particularly in rural areas.

In rural areas, it will be different. Patients still want to see ‘the doctor.’ That belief will take time to change. – HM 4

Participants suggested that trust would grow once patients experience the competence of APNs firsthand.

#### Collaboration and interdisciplinary teamwork

APNs were envisioned as collaborative partners in care, working with physicians, lifestyle specialists, and other healthcare professionals.

The APN can create a care plan with the family doctor, monitor the patient’s indicators, and coordinate with the team. – HM 2

Interdisciplinary trust and communication were viewed as essential for successful integration. Some healthcare managers proposed structured collaboration strategies, including joint training and informal team building.

Unite everyone into one team to work cooperatively… training or after-work events could improve relationships. – HM 3

Others suggested a phased approach—beginning with shared tasks and transitioning toward independent APN-led care.

### Long-term vision and impact on the healthcare system

Most HMs expressed optimism that APNs would contribute meaningfully to the future of Lithuanian healthcare. Their introduction was seen as inevitable and essential for improving service delivery, especially in response to aging populations and rising multymorbidity.

APNs will be employed according to need and will take over tasks from family doctors and GPNs… access to services should improve markedly. – HM 2

APNs are practically like doctors… with time, the population will accept and even prefer them for certain cases. – HM 4

HMs highlighted the importance of professional respect, visibility, and long-term policy support to sustain these roles.

### Defining successful APN integration

Success was defined by clarity, trust, and autonomy. HMs felt that legal certainty, fair compensation, public education, and institutional support were key pillars of sustainable APN integration.

Success means allowing each specialist to work fully within their competencies and have autonomy. – HM 2

Public education about the new service is key. – HM 1

It would mean having a defined and recognized APN role, supported by national legislation, funded training programs, and measurable outcomes. – HM 6

Some referenced the historical role of feldshers and nurses with extended responsibilities as a precedent, suggesting that APNs represent a formal and modern evolution of this concept.

Compared to other countries, we’re far behind… APNs are a modern version of feldshers—independent, community-focused, and practical. – HM 3

These expanded findings illustrate a cautious yet hopeful stance from healthcare managers. They largely align with the aspirations of APNs themselves and provide clear strategic directions for successful integration—emphasizing collaboration, regulation, education, and structural investment.

### Comparative perceptions of APN integration

#### Understanding of APN role

Nurses see themselves as still working within general nursing roles but expect to gain more autonomy and clinical responsibility as APNs are integrated. Healthcare managers describe APNs as independent clinicians who have the potential to manage patients and make decisions on their own.

#### Contribution to healthcare

Nurses believe that APNs will strengthen chronic care management, reduce wait times, and improve healthcare accessibility, particularly in rural areas. In comparison, healthcare managers see APNs as a way to alleviate doctors’ workloads and broaden patient access, especially in underserved communities.

#### Barriers to integration

Nurses highlight the absence of clear legal frameworks, resistance from colleagues, and poorly defined roles as the main barriers to APN integration. In comparison, healthcare managers emphasize regulatory uncertainty, interprofessional skepticism, and insufficient funding as the primary challenges to successful implementation.

#### Support and resources needed

Nurses stress the importance of mentorship, dedicated APN training programs, and strong policy-level backing to ensure successful integration. In contrast, healthcare managers emphasize the need for well-defined job descriptions, sufficient funding, appropriate infrastructure, and robust administrative support to facilitate the effective implementation of the APN role.

#### Future vision and system impact

Nurses envision APNs as leaders in public health, patient educators, and autonomous care providers delivering advanced clinical services. Meanwhile, healthcare managers anticipate a transformation in care models, greater system efficiency, and APNs filling diverse roles across departments such as primary care, chronic disease management, and emergency care.

#### Definition of successful integration

Nurses stress the importance of legal recognition, increased public awareness, and the creation of structured APN positions that align with their specialized training. In comparison, healthcare managers emphasize the need for clearly defined competencies, greater clinical autonomy, measurable outcomes, and strengthened professional respect to ensure successful APN integration.

## Discussion

The findings indicate readiness, motivation, and emerging role clarity among Lithuanian APNs in relation to transforming healthcare delivery; however, these results should not be interpreted as representative of all advanced practice nurses in Lithuania. While siginificant challenges remain, there is a clear consensus among both APNs and healthcare managers on the need for coordinated institutional, legal, and cultural change to unlock the full potential of advanced nursing practice. This study contributes novel insights into how APN integration is perceived in Lithuania and situates these findings within the broader international context.

Consistent with international evidence, Lithuanian APNs reported that they continue to perform primarily general nursing tasks despite holding master’s-level qualifications and expressing readiness for greater autonomy. Similar patterns have been observed in other countries where role development has outpaced regulatory reform. For example, Hill et al. (2023) found that unclear role definitions constrained community APNs in the United Kingdom [25], while Beil-Hildebrand and Smith (2022) described comparable limitations in German-speaking countries linked to hierarchical professional cultures [50]. In Lithuania, healthcare managers acknowledged the clinical potential of APNs but emphasized the absence of a clear legislative framework—echoing the International Council of Nurses’ position that the lack of regulation remains a fundamental barrier to APN practice [51].

Both participant groups identified physician shortages, increasing chronic disease burden, and limited access to care in rural areas as key drivers for APN integration. These findings align closely with the Lithuanian Ministry of Health’s *Action Plan to Reduce the Shortage of Doctors* [28]. International experience demonstrates that APNs are well positioned to address such systemic pressures. In France and Spain, for instance, APNs have improved access to care and continuity in underserved areas, particularly through expanded roles in chronic disease management and patient education [12, 14, 20].

Healthcare managers consistently highlighted the importance of clearly defined job descriptions, legal authority, and appropriate remuneration. While these issues were acknowledged previously, the present findings underscore that reimbursement and funding mechanisms are not merely technical considerations but central implementation levers for APN integration. International studies indicate that APN integration is most successful when financing models explicitly recognize advanced competencies and professional autonomy. Toniolo et al. (2024) demonstrated that alignment between regulation, funding, and organizational readiness was crucial for APN role consolidation in France [51]. Unsworth et al. (2024) similarly emphasized that sustainable funding, leadership engagement, and transparent career pathways are essential to retaining APNs and preventing role erosion [52].

In the Lithuanian context, the absence of nationally defined reimbursement schemes risks perpetuating situations in which APNs perform advanced tasks without formal recognition or compensation. This mismatch between responsibility and reward may contribute to professional dissatisfaction and burnout—an association well documented in international literature. Colson et al. (2021) reported increased burnout risk among APNs working in under-regulated systems, particularly where expectations exceed formal authority and remuneration [53]. Addressing reimbursement and funding structures is therefore not only a workforce issue but also a patient safety and system sustainability concern.

Effective collaboration with physicians emerged as another critical factor. Participants described trust, role clarity, and shared responsibility as prerequisites for successful interprofessional practice. These findings are consistent with Lee et al. (2023), who showed that early clarification of role boundaries facilitates smoother APN integration [35], and with Torrens et al. (2020), who identified physician resistance and unclear accountability as persistent barriers across Europe [19]. The Lithuanian findings further suggest that, in the absence of formalized frameworks, APNs risk being perceived either as “super-assistants” or informal physician substitutes rather than as autonomous professionals.

Public and professional awareness of APN roles in Lithuania remains limited. As shown by Darginavičienė et al. (2022), patients often lack clarity about when and why to consult an APN [24]. By contrast, countries such as the United Kingdom and the Nordic states have invested in public education campaigns and clearly structured APN services, leading to higher acceptance and utilization [4, 54]. Lithuanian APNs demonstrated strong intrinsic motivation to expand their roles, similar to Finnish APNs whose professional identity is closely linked to autonomy and clinical responsibility [22]. However, sustained motivation is contingent on visible institutional support, clear career trajectories, and equitable compensation.

Importantly, the findings indicate that while national legislation is essential, meaningful progress can begin before formal regulatory reform is completed. Drawing on international experience, practice-oriented and transitional strategies may support implementation in the interim, including locally agreed protocols that define APN responsibilities, pilot projects with clearly monitored outcomes, and formalized supervision and collaboration agreements with physicians. Such transitional approaches have been successfully used in countries like France and Spain to test APN roles, generate implementation evidence, and inform national policy decisions [14, 20, 51].

Based on the findings and international evidence, the following recommendations emerge:

- Clarify APN roles through national legislation, supplemented by interim local protocols while awaiting legal reform [5, 14].
- Establish sustainable reimbursement and funding mechanisms that explicitly reflect APN competencies, autonomy, and responsibility [51, 52].
- Strengthen interprofessional collaboration through clear role boundaries, supervision models, and joint training [35].
- Increase public and professional awareness of APN roles to enhance acceptance and utilization [24, 54].
- Expand and systematically evaluate pilot projects to build local evidence for national rollout [20, 51].

Overall, Lithuania shares many of the challenges encountered by countries at earlier stages of APN development but also has the opportunity to learn from established European models. A coherent, framework-guided approach—such as that offered by the PEPPA framework [7] —combined with targeted investment in education, regulation, and financing, may enable Lithuania to harness the full potential of Advanced Practice Nursing and strengthen its healthcare system in a sustainable and patient-centred manner.

## Conclusions

This study highlights that within the study cohort, both Advanced Practice Nurses and healthcare managers perceive APN integration as an important and promising development for the Lithuanian healthcare system. Participants identified potential roles for APNs in supporting primary care, contributing to chronic disease management, and alleviating pressures associated with physician shortages, particularly in underserved areas. However, these views should be understood as anticipated benefits rather than demonstrated outcomes, as the study explored perceptions rather than clinical, organizational, or economic effects.

APNs participating in the study expressed strong motivation to expand their roles, yet also reported uncertainty linked to unclear institutional support and poorly defined role boundaries. Healthcare managers similarly emphasized that current barriers to integration are primarily structural and cultural, including the absence of clear legislation, uncertain funding mechanisms, limited public awareness, and resistance within professional hierarchies. While participants believed these challenges could potentially be addressed through policy development, education, and interprofessional collaboration, the feasibility and effectiveness of such measures require further empirical investigation.

At an early stage of APN development, Lithuania appears to be navigating challenges similar to those encountered by other European countries prior to formal role consolidation. The findings suggest a perceived opportunity—rather than a guaranteed outcome—to advance APN roles through coordinated efforts in education, regulation, and organizational support, informed by international experience and best practices.

Change management in healthcare is inherently gradual, and APN integration should be viewed as a progressive process rather than a single solution to workforce or system-level challenges. Overall, the perspectives captured in this study underscore the need for clearer legal frameworks, well-defined professional roles, and sustained institutional support to enable the meaningful integration of APNs in Lithuania. Further research involving larger samples and outcome-focused designs is necessary to evaluate the impact of APN integration on patient care, workforce sustainability, and health system performance.

## Supplementary Information

Below is the link to the electronic supplementary material.

Supplementary Material 1

## Data Availability

All data generated or analysed during this study are included in this published article. The data supporting the findings of this study are available from the corresponding author upon reasonable request.

## Declarations

### Authors information

IQ is an Advanced Practice Nurse, MSc, and was a master’s student during the data collection phase at the Faculty of Medicine of Vilnius University, Lithuania. JS holds her PhD in Health Sciences and serves as an docent at Vilnius University, Lithuania. NI holds her PhD in Health Sciences and contributes as a Professor and Senior Researcher at the Vilnius University, Lithuania.

## Abbreviations

APN: Advance practice nurse
ICN: International Council of Nurses
HM: Healtcare manager
NP: Nurse Practitioner
RN: Registered nurses
