# Supplementary material for: Integrating advanced practice nurses into the health system: views from nurses and healthcare managers – a qualitative study
Source: BMC Nurs. 2026 Feb 20;25:283. doi: 10.1186/s12912-026-04427-z (PMC13032527; doi:10.1186/s12912-026-04427-z)
Supplement: Supplementary file 1 — Supplementary Material 1 [file 12912_2026_4427_MOESM1_ESM.docx]

## **Semi-Structured Interview Guide for Advanced Practice Nurses (APNs)**

**1. Understanding Current Roles and Experiences, Perceived Need for Advanced Practice Nurses**

- Can you describe your current role and responsibilities within your organization?
- How do you see your role evolving with the integration of APNs into the Lithuanian health system?
- What challenges have you faced in your nursing practice that you think APNs could help address?
- In your opinion, what are the main healthcare needs in Lithuania that APNs could address?
- How do you think APNs could impact patient outcomes or the overall quality of care?

**2. Motivations and Interest in Advanced Practice Nursing, Perceived Challenges and Barriers to APN Integration**

- What motivates you to pursue or support advanced practice roles within the nursing profession?
- How do you think the integration of APNs could enhance your own professional development and career satisfaction?
- What challenges do you foresee with integrating APNs into the Lithuanian health system?
- What would need to change within the current healthcare system to successfully integrate APNs?

**3. Support and Resources for Successful APN Integration, Expectations and Potential Impact on the Health System**

- What support or resources do you think are necessary for APNs to succeed in the healthcare system?
- How could healthcare organizations, educational institutions, or policymakers better support nurses in transitioning to APN roles?
- What impact do you expect the integration of APNs to have on the Lithuanian health system as a whole?
- What would a successful integration of APNs look like to you?
- Is there anything else you’d like to add about how APNs could contribute to improving healthcare in Lithuania?

## **Semi-Structured Interview Guide for Healthcare managers**

**1. Perception of Advanced Practice Nursing Role**

- What is your understanding of the role of Advanced Practice Nurses (APNs) in the healthcare setting?
- How do you perceive the potential contributions of APNs to patient care within this hospital/primary health care centre?
- Are there specific areas or departments where you feel APNs could make a significant impact?

**2. Need and Value of APN Integration**

- In your view, what are the main needs in the healthcare system that APNs could help address?
- How do you think integrating APNs could contribute to meeting organizational goals, such as improving patient outcomes and ensuring patient safety or reducing wait times?

**3. Implementation, Support, Cost, Funding, and Financial Considerations**

- What are some of the key resources or support mechanisms needed for the successful integration of APNs?
- What challenges do you anticipate in integrating APNs into your current organizational structure?
- Are there specific changes to health policies, staffing models, or organizational practices that you think would facilitate APN integration?
- How would the integration of APNs affect the hospital’s budget or resource allocation?
- Do you foresee any financial challenges or benefits associated with employing APNs?
- Are there funding or reimbursement considerations that need to be addressed for the sustainable integration of APNs?

**4. Expectations for Interdisciplinary Collaboration**

- How do you envision APNs working with physicians and other healthcare professionals within your hospital?
- Do you anticipate any challenges or resistance in fostering collaboration between APNs and other staff?
- What strategies would you consider enhancing interdisciplinary collaboration involving APNs?

**5. Long-term Vision and Impact on Healthcare Delivery**

- How do you see APN integration shaping the future of healthcare delivery in this hospital or in Lithuania more broadly?
- What would a successful integration of APNs look like from your perspective?
- Is there anything else you’d like to add about the potential impact or value of APNs in the Lithuanian healthcare system?
